# Optimizing Tonpilz Transducer Transmission Through Impedance Matching and Head Mass Structure

**DOI:** 10.3390/mi16030352

**Published:** 2025-03-20

**Authors:** Yang Gou, Shenhai Ye, Xin Fu, Fanghua Zheng, Xuzhong Zha, Cong Li

**Affiliations:** 1 College of Logistic Engineering, Shanghai Maritime University, Shanghai 201306, China; 2 Nantong Chaolin Intelligent Technology Co., Ltd., Nantong 226010, China; 3 College of Information Engineering, Shanghai Maritime University, Shanghai 201306, China; 4 Chongqing Innovation Center, Beijing Institute of Technology, Chongqing 401151, China; 5 School of Information and Electronics, Beijing Institute of Technology, Beijing 100081, China

**Keywords:** Tonpilz transducer, electrical impedance matching, head mass structure, broadband high-power excitation

## Abstract

The bandwidth and output power of underwater acoustic transmitters are important for high-performance sonar detection systems. A mismatch between the impedance of the transducer and the transmitting circuit results in a low power factor, significantly limiting the sonar’s operating bandwidth and detection range. In addition, the radial head structure of the Tonpilz transducer plays an important role in determining the radiation characteristics of the sound field. This paper proposes a new radiation head structure along with an impedance-matching network circuit. First, a mathematical model of active power is established based on the Krimholtz–Leedom–Matthaei (KLM) model of the transducer. The adaptive Gauss–Newton algorithm is then used to calculate the parameters of the broadband impedance-matching network components, ultimately determining the network parameters and the structure of the transducer’s radiation head. Experimental results indicate that the transmitter voltage response of the proposed transducer is 6 dB higher than that of a conventional transducer and can be further increased by 5 dB with impedance matching. The impedance-matching network enhances the power factor of the transducer by 3.2 times, expands the frequency band by a factor of 1.6, and significantly enhances the acoustic field radiation characteristics of the underwater acoustic transducer.

## 1. Introduction

Underwater acoustic transducers are essential technical equipment for ocean exploration and engineering, with their performance directly determining the quality of underwater acoustic detection. Tonpilz acoustic emission transducers offer advantages such as high power, stable performance, easy array deployment, compact size, and high sensitivity. These features make them widely used in various fields, including underwater communication, positioning and navigation, marine resource assessment, and unmanned underwater vehicle (UUV) search and rescue operations [1,2,3]. With advancements in underwater acoustic technology and growing demand in both civil and military applications, the performance requirements for underwater acoustic transducers have significantly increased. These transducers must now handle higher volumes of data transmission, which is directly affected by the working bandwidth of composite rod transducers. To achieve higher communication quality, the transducers must possess a sufficiently broad working bandwidth and strong radiation energy to transmit more information effectively [4,5]. Tonpilz transducers are typically capacitance-loaded and exhibit multiple resonant frequencies with sharp impedance fluctuations. Their impedance characteristics are difficult to express through specific analytical functions, posing challenges such as an unclear frequency response of the excitation signal, complexity in matching network topology, and difficulty in converging multi-component parameter calculations. These challenges are key bottlenecks in broadening the working frequency band of transducers [6,7]. Currently, most optimization efforts focus separately on electrical impedance matching and acoustic field radiation characteristics, without integrating these aspects in the structural optimization of transducers. This lack of integration limits the overall performance improvements achievable for underwater acoustic transducers.

In terms of transducer excitation characteristics, Wang Jun et al. studied a thin circular tube transducer for acoustic logging applications. Using wave and piezoelectric equations, they modeled the transducer’s mechanical vibration, analyzed its vibration response under sinusoidal, pulse square, and triangular wave excitations, and selected the optimal excitation function according to specific borehole parameters. This study serves as an effective reference for analyzing the vibration response of transducer excitation signals [7,8,9]. However, the thin circular tube transducer is not applied to underwater acoustics, and its mechanical model overlooks the influence of radial stress while failing to integrate the electrical and acoustic characteristics of the transducer. As a result, it is challenging to determine which component parameters are critical for improving power and bandwidth. Fallin et al. established the electroacoustic conversion function for spherical transducers, describing the electroacoustic energy conversion process; however, their work did not combine the analysis of frequency band characteristics with impedance-matching calculations [10,11]. Regarding broadband characteristics, most studies focus on material and structural optimization. Yongrae designed a cavity head mass wideband Tonpilz transducer to broaden the bandwidth by enhancing coupling between the longitudinal mode of the transducer and the patting mode of the head mass. This design achieved a lower mechanical quality factor and a bandwidth of up to 129.5% at −6 dB [12]. Zhou developed a microstructure to fabricate a conductive oxide LaNiO_3_ (LNO) thin film approximately 300 nm thick on an insulating silicon substrate using the metal–organic decomposition (MOD) method. A randomly oriented PZT film with a thickness of approximately 7 µm was then deposited on the LNO-coated SOI substrate using an improved sol-gel process for underwater acoustic transducer applications [13,14,15].

Although incorporating a hole inside the mass block at the front of the transducer can improve bandwidth, studies on its directivity remain limited, and alternative effective structures have not been extensively explored [16,17,18]. When designing the head mass block of the transducer, its weight significantly affects the frequency characteristics, complicating the design process and potentially degrading the transducer’s original performance. Therefore, it is essential to develop a new structure that broadens the working bandwidth while minimizing the impact on frequency response. Conventional methods for bandwidth broadening mainly focus on optimizing the design of the acoustic matching layer. However, this approach restricts the transducer’s application scenarios. When the external medium load changes, the acoustic matching layer becomes ineffective and no longer applicable [19,20,21,22,23].

In this study, the conventional Tonpilz transducer’s head mass was modified, and an impedance-matching network was integrated. Experimental results show that the transmitter voltage response of the novel transducer is 6 dB higher than that of a conventional transducer, with an additional improvement of 5 dB after impedance matching. The active power of the transducer increased by 3.5 times, and the frequency band was broadened by a factor of 1.5. Hydrophone and directivity tests confirmed that the proposed method effectively enhances the sound field radiation characteristics of the Tonpilz hydroacoustic transducer.

## 2. Theoretical Analysis of Broadband Impedance-Matching System for Novel Tonpilz Transducer

**Figure 1:**
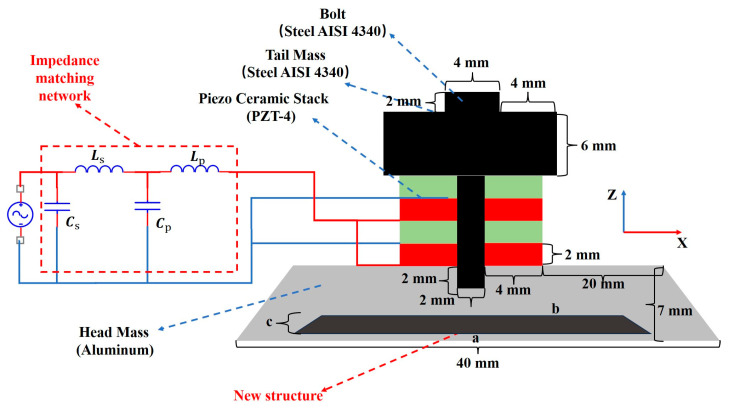
New Tonpilz transducer planar structure and impedance-matching network. The red part is the positive voltage connection port of the transducer, and the blue part is the negative voltage port. “a” represents the bottom length of the filling structure, and “b” represents the top length of the filling structure.

A piezoelectric transducer used in underwater detection equipment is an electromechanical device, with its mechanical vibration characteristics directly determining its acoustic field radiation performance. The X-Z projection of the Tonpilz transducer used in this study is shown in Figure 1, comprising bolts, a tail mass, a piezoceramic stack, and a head mass. The four adjacent piezoelectric ceramics have opposite polarization directions, but the applied voltage direction remains the same, ensuring that all piezoelectric rings vibrate in phase to achieve maximum driving efficiency. A π-type impedance-matching network is installed at the excitation end of the transducer. Additionally, a slot is cut into the front mass block, and a solid filler is added to improve the sound field radiation characteristics. Based on the improved Krimholtz–Leedom–Matthaei (KLM) model, the parameter calculation methods for the impedance-matching network and the filling structure are subsequently explored.

**Figure 2:**
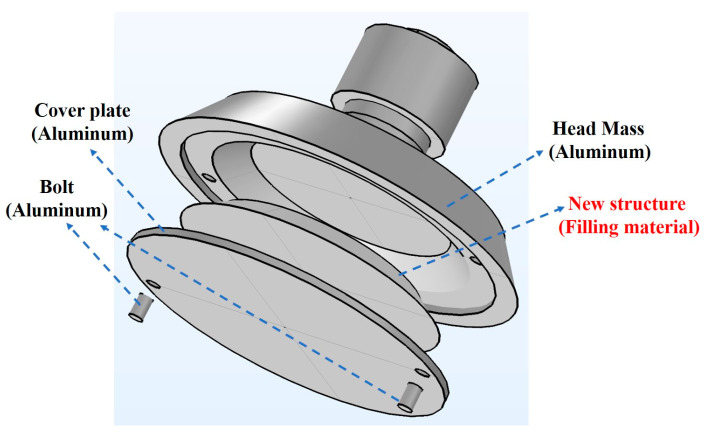
3D assembly diagram of the transducer.

Figure 2 shows the 3D assembly diagram of the new transducer. The filling structure is located inside the front mass block and bolted to a base plate made of the same material. This design allows the filling material to be adjusted at any time according to the operating environment, ensuring that the transducer maintains optimal output performance. To obtain faster vibration speeds, the front mass is made of lightweight duralumin, while the assembly base plate and bolts are also constructed from duralumin. The optimization of the filling material and structural parameters is discussed in detail later.

**Figure 3:**
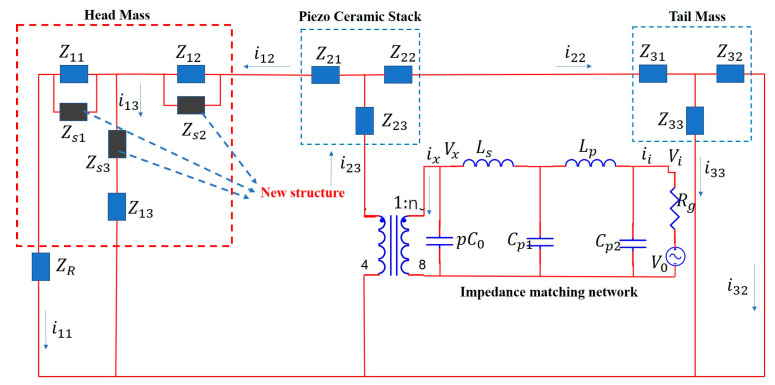
KLM model of the improved Tonpilz transducer.

To integrate the parameter optimization of the front mass block with the impedance-matching network calculation, a broadband impedance-matching network was introduced based on the conventional KLM model [24,25]. Unlike traditional designs, the new structure proposed in this study does not include an additional matching layer. Instead, a groove is cut inside the front mass block to accommodate different filling materials, transforming the sound field radiation characteristics. In the equivalent circuit, the impedances $Z_{s1}$ and $Z_{s2}$ are connected in parallel with the original model, while the remaining impedance $Z_{s3}$ is in series with the radiating head model. This configuration enables the adjustment of filling material parameters to modify mechanical properties such as Young’s modulus and density according to specific requirements. To simplify theoretical calculations, the geometric structure of the filling material in this paper is identical to that of the original mass blocks. Figure 3 shows the improved KLM model of the Tonpilz transducer, which adds a broadband impedance-matching network and a newly designed front mass block, building upon the conventional model.

As shown in Figure 3, $Z_{ij}$(*i* = 1, 2, 3; *j* = 1, 2, 3) represents the mechanical impedance of each part [26], where:

(1) $$Z_{11}=-j\rho_{2}C_{2}S_{1}(\frac{1}{k_{2}L_{2}}\left(\sqrt{\frac{S_{2}}{S_{1}}}-1\right)+\cot\left(k_{2}L_{2}\right)-\frac{\sqrt{S_{1}S_{2}}}{S_{1}\sin\left(k_{2}L_{2}\right)})$$

(2) $$Z_{12}=-j\rho_{2}C_{2}S_{2}(\frac{1}{k_{2}L_{2}}\left(\sqrt{\frac{S_{1}}{S_{2}}}-1\right)+\cot\left(k_{2}L_{2}\right)-\frac{\sqrt{S_{1}S_{2}}}{S_{2}\sin\left(k_{2}L_{2}\right)}$$

(3) $$Z_{13}=\frac{\rho_{2}C_{2}\sqrt{S_{1}S_{2}}}{\mathrm{jsin}\left(k_{2}L_{2}\right)}$$

In Equations (1)–(3), $\rho_{2}$ is the density of the head mass, $C_{2}$ is the sound velocity of the head mass, $S_{1}$ is the bottom area under the head mass platform, n is the mechanical coupling coefficient, and $S_{2}$ is the top area on the head mass platform. $L_{2}$ and $k_{2}$ are the thickness and wave number of the head mass material, respectively. The transformer in Figure 3 represents the coupling relationship only; the actual current flows through it.

(4) $$Z_{21}=Z_{22}=j\rho_{3}C_{3}S_{3}\tan\left(\frac{pk_{3}L_{3}}{2}\right),\;\;\;\;Z_{23}=\frac{\rho_{3}C_{3}S_{3}}{jsin(pk_{3}L_{3})}$$

(5) $$C_{0}=\frac{S_{3}\varepsilon_{33}^{T}S_{33}^{D}}{L_{3}S_{33}^{E}},\;n=\frac{S_{3}d_{33}}{L_{3}S_{33}^{E}}\;$$

In Equation (4), $\rho_{3}$, $C_{3}$, $S_{3}$, $k_{3}$, $L_{3}$, and p are the density, sound velocity, cross-sectional area, wave number, thickness, and number of piezoelectric ceramic materials, respectively. In Equation (5), $\rho_{4}$, $C_{4}$, $S_{4}$, $k_{4}$, $L_{4}$, and p are the density, sound velocity, cross-sectional area, wave number, thickness, and number of tail mass, respectively.

(6) $$Z_{31}=Z_{32}=j\rho_{4}C_{4}S_{4}tan\left(\frac{k_{4}L_{4}}{2}\right),\;Z_{33}=\frac{\rho_{4}C_{4}S_{4}}{jsin(pk_{4}L_{4})}$$

(7) $$Z_{R}=\rho_{w}C_{w}S_{1}[1-\frac{J_{1}(2k_{w}a)}{k_{w}a}+\frac{jS_{1}(2k_{w}a)}{k_{w}a}]$$

Here, $Z_{R}$ is the transducer load impedance, where $\rho_{w}$, $C_{w}$, and $k_{w}$ are the density, sound velocity, and wave number of water, respectively, while *a* denotes the radius of the radiation surface. In Equation (8), $\rho_{s}$, $C_{ss}$, $S_{s1}$, $S_{s1}$, $l_{p}$, and $k_{p}$ are the density, sound velocity, bottom area, top area, thickness, and wave number of the filling material, respectively.

(8) $$Z_{s1}=Z_{s2}=\frac{\rho_{s}C_{ss}S_{s1}}{j}\tan\left(\frac{k_{p}l_{p}}{2}\right),\;Z_{s3}=\frac{{j\rho }_{s}C_{ss}\sqrt{S_{s1}S_{s2}}}{sin(k_{p}l_{p})}$$

The parameters of the broadband impedance-matching network were calculated based on this model.

## 3. Study on Filling Material Parameters of New Tonpilz Transducer

Before designing the impedance-matching network, it is necessary to determine the material and structural parameters of the filler. The fixed parameters of the transducer are shown in Figure 1. The material and structural parameters of the filling part are discussed below. The main material parameters of the transducer are shown in Table 1. The filling materials for the transducer are mainly epoxy resin and steel. Table 2 shows the materials and geometric initialization parameters of the filling structure.

**Figure 4:**
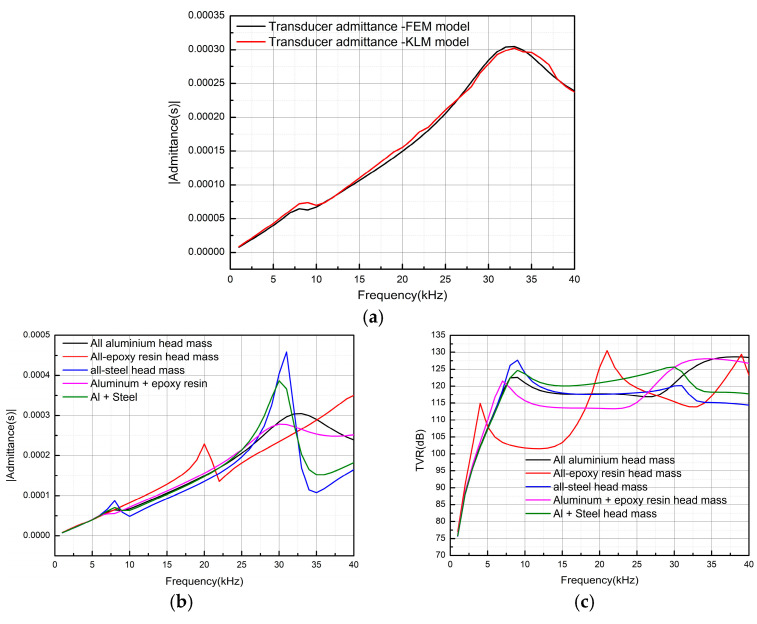
Transducers’ admittance and TVR obtained for head masses composed of different materials. (**a**) Comparison of admittance between KLM model and FEM model, (**b**) admittance, (**c**) TVR.

First, it is necessary to determine the appropriate filling structural materials and analyze them using the finite element method (initialization state: a = 36 mm, b = 24 mm, c = 3 mm in the filling structure). Figure 4 compares the admittance and transmitting voltage response (TVR) curves of the transducer head mass for different material combinations and shows the comparison of admittance between the FEM model and KLM model of transducers. In Figure 4a, the admittance comparison between the transducer FEM and the KLM model is shown. It can be seen that the two curves fit well on the whole. At the resonant frequency of 7.5 kHz, the admittance value of the KLM model is higher, and there is a certain deviation between the two curves. Therefore, the analytic solution can converge better. In general, the KLM model can describe the admittance characteristics of the transducer in detail, which lays a good theoretical basis for the subsequent impedance matching. In Figure 4b, it can be seen that, except for epoxy resin, other materials have little influence on the resonant frequency. The resonant frequency of the transducer is approximately 7.5 kHz and 30 kHz. The admittance values for all-steel and aluminum–steel composite materials are the highest, indicating that transducers made from these materials exhibit the best electrical characteristics at the resonant frequency. Figure 4c shows that the TVR of the transducer head mass made from composite structures and materials is superior to that of a single material. This is particularly evident in the case of steel combined with aluminum, which significantly improves the TVR. When aluminum and steel are used as the filling materials, the TVR of the transducer increases by 13 dB, with a marked improvement in flatness. In contrast, all-aluminum and all-steel materials have minimal influence on the TVR. To obtain higher vibration speed, aluminum is typically selected as the head mass material for Tonpilz transducers. However, combining aluminum and epoxy resin results in a 5 dB increase in TVR within the 7.5–30 kHz frequency range.

**Figure 5:**
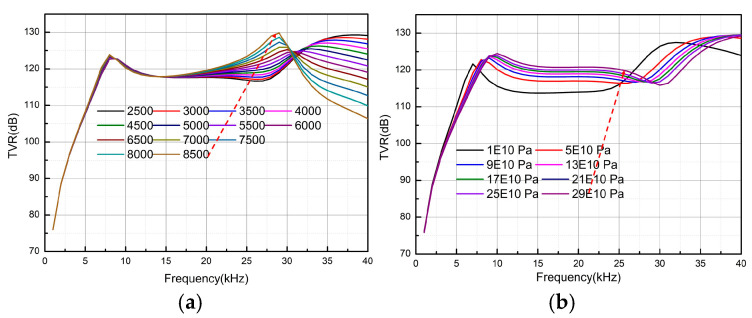
TVR of the head mass filling structure under different densities and Young’s moduli. (**a**) Effect of density on TVR, (**b**) effect of Young’s modulus on TVR.

Based on the above analysis, it can be concluded that the head mass with the filler structure provides the best performance, offering a high TVR while meeting the requirements for resonant frequency and frequency band flatness. As shown in Table 2, the Poisson’s ratios of the three materials are similar, while their densities and Young’s moduli differ significantly. As shown in Figure 5, a parametric scanning analysis was performed with the Young’s modulus fixed at 70 GPa and the density at 2500 $kg/m^{3}$.

**Figure 6:**
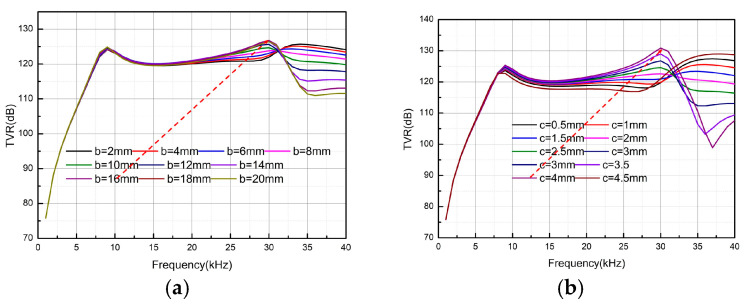
TVR of the head mass filling structure with different lengths and thicknesses. (**a**) Effect of length on TVR, (**b**) effect of thickness on TVR.

Considering TVR as the standard, Figure 5a shows that the transmitting voltage response is positively correlated with the density, while the flatness in the frequency band remains relatively unaffected. However, both parameters reverse their trends at approximately 30 kHz. In Figure 5b, Young’s modulus is also positively correlated with the transmitting voltage response, but after 30 kHz, the assigned value increases rather than decreases. In summary, a filling material with high density and a high Young’s modulus should be selected. To facilitate material selection and processing, this paper uses steel (as listed in Table 2) as the filling material. The next step involved further analysis of the size parameters. In optimizing the structural parameters, the bottom radius of the fixed filling structure was set at 28 mm, and the height and top radius were analyzed. As shown in Figure 6a, the height of the circular table was fixed at 4 mm, while the radius of the top surface was varied from 2 mm to 20 mm in 2-mm steps. The change in TVR in the 5–30 kHz frequency band is positively correlated with the radius of the top surface, reaching its maximum when the radius of the top surface is close to that of the bottom surface. Therefore, 16 mm was chosen as the optimal radius for the top surface. In Figure 6b, the bottom and top radii were set at 18 mm and 16 mm, respectively, while the thickness was varied from 0.5 mm to 4.5 mm in 0.5-mm steps. Initially, the TVR increases with thickness, reaching its peak at 4 mm. The TVR then decreases sharply, with the lowest value at 4.5 mm. Based on the above analysis, the optimal structural parameters for the filling material were determined as a = 18 mm, b = 16 mm, c = 4 mm.

**Figure 7:**
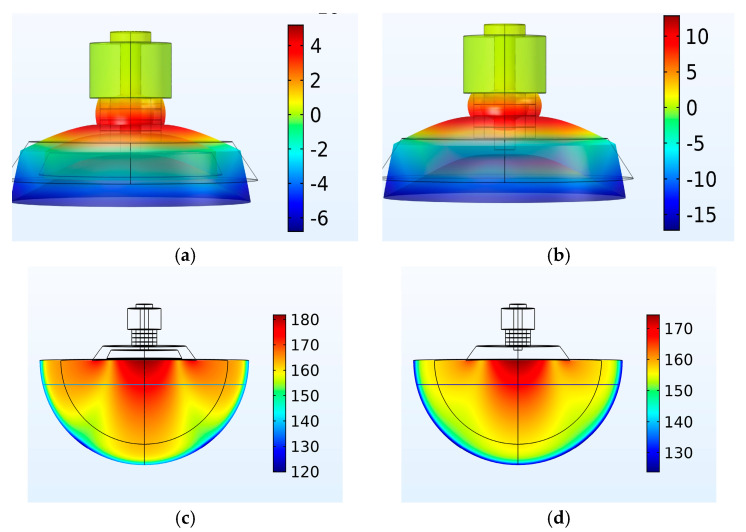
Vibration velocity and sound field radiation characteristics of the radiation head of the new transducer and conventional transducer. (**a**) Vibration velocity of the head mass of the new transducer—30 kHz; (**b**) head mass vibration velocity of the conventional transducer—30 kHz; (**c**) new transducer sound pressure level—30 kHz; (**d**) sound pressure level of the conventional transducer—30 kHz.

Next, the longitudinal vibration velocity of the transducer at 30 kHz and the water-radiated sound pressure level were used as references to compare and analyze the conventional and new Tonpilz transducers of the same material and size. As shown in Figure 7a,b, the longitudinal vibration velocity of the head mass of the new transducer is four times that of the conventional transducer. In Figure 7c,d, the radiated sound pressure level at the center of the new transducer increased by 10 dB compared to the conventional transducer.

Based on the finite element analysis of the transducer, it can be concluded that the new transducer designed in this paper offers significant advantages in acoustic field radiation characteristics compared to the conventional transducer. An excitation circuit experiment will be carried out in future work.

## 4. Broadband Impedance-Matching Network Parameter Calculation

The traditional method for calculating the parameters of a transducer’s wideband impedance-matching network elements is based on the real-frequency data method. The network transfer function is calculated according to the actual impedance data of the transducer so that the imaginary part of the system impedance is reduced to zero and the real part is close to the output resistance of the signal source. This method is typically used for large hydroacoustic transducers with relatively flat admittance frequency responses. However, it is less effective for small transducers with sharply fluctuating impedance. In addition, the Butterworth–Van Dyke (BVD) equivalent circuit model struggles to accurately represent the electromechanical coupling characteristics of the transducer, making it difficult to correlate electrical and acoustic characteristics and jointly optimize material and structural parameters [27,28]. The enhanced KLM model proposed in this paper effectively combines impedance-matching network parameter calculation and transducer parameter optimization, offering a more intuitive and simplified approach.

Based on the impedance distribution shown in Figure 3, the impedance function of the model system was derived using Kirchhoff’s voltage and current laws:

(9) $$j\omega L_{s}i_{i}+{\left(j\omega C_{0}\left(C_{P2}+C_{P1}\right)\right)}^{-1}\left(i_{i}-i_{x}\right)=V_{i}$$

(10) $$j\omega L_{p}i_{x}+{\left(j\omega C_{0}\right)}^{-1}\left(i_{x}ni_{23}\right)={\left(j\omega C_{p2}\right)}^{-1}\left(i_{i}-i_{x}\right){\left(j\omega C_{p1}\right)}^{-1}$$

(11) $$V_{x}=HV_{i}-j\omega (L_{s}i_{x}+L_{p}i_{i})$$

(12) $$i_{x}=ni_{23}+V_{x}{\left(j\omega C_{0}\right)}^{-1}$$

(13) $$\frac{(\frac{Z_{12}+Z_{s2}}{Z_{12}Z_{s2}}+\frac{Z_{11}+Z_{s1}}{Z_{11}Z_{s1}})i_{13}}{Z_{13}+Z_{s3}}+Z_{R}i_{11}=nV_{x}-Z_{23}i_{23}$$

According to Equations (9)–(13), the following expression is obtained:

(14) $$Z=\frac{V_{i}}{i_{i}}=\frac{Q+j\left(G-jn^{2}\left(L_{p}+L_{s}\right)\omega \right)C_{p0}+j(C_{p2}C_{p1}C_{p0}L_{s}\omega^{2}-1))L_{p}}{H+\left(-\omega^{3}C_{p1}C_{p2}L_{p}^{2}+jn^{2}\omega^{2}C_{p1}C_{p2}L_{p}+\omega \left(C_{p0}+L_{p}\right)-jn^{2}\right)C_{0}+C_{p1}C_{p2}C_{p0}L_{p}\omega }$$

The impedance expression for the entire model is given by:

(15) $$Z=\frac{V_{i}}{i_{i}}=\frac{Q+j\left(G-jn^{2}\left(L_{p}+L_{s}\right)\omega \right)C_{p0}+j(C_{p2}C_{p1}C_{p0}L_{s}\omega^{2}-1))L_{p}}{H+\left(-\omega^{3}C_{p1}C_{p2}L_{p}^{2}+jn^{2}\omega^{2}C_{p1}C_{p2}L_{p}+\omega \left(C_{p0}+L_{p}\right)-jn^{2}\right)C_{0}+C_{p1}C_{p2}C_{p0}L_{p}\omega }$$

where:

(16) $$Q=\frac{{(Z}_{13}+Z_{s3})(Z_{s1}+Z_{s2})(Z_{23}+Z_{31})(Z_{33}+Z_{32})(Z_{11}(Z_{12}+Z_{13}+Z_{21}{)+Z}_{13}(Z_{12}+Z_{21}))}{Z_{R}}$$

(17) $$H=\left(\left(Z_{13}+Z_{12}+Z_{s1}+Z_{23}\right)Z_{33}+\left(Z_{22}+Z_{23}+Z_{31}\right)\left(Z_{s2}+Z_{13}+Z_{21}\right)\right)Z_{R}\;+Z_{33}(Z_{22}+Z_{23}+Z_{31})(Z_{12}{+Z}_{13}+Z_{s3})$$

In this paper, the impedance at the load end of the impedance-matching network and the transducer KLM model was limited to a certain range to meet the bandwidth requirements for calculating network parameters. Since singularities were encountered during the calculation, initial values were selected for the first test. If a non-convergent singularity was detected, all boundary conditions in the algorithm were adjusted and guided to the convergence interval before starting the computation task. The specific numerical filtering equation is as follows:

(18) $$X_{i,j}\left(t+1\right)=\left\{\begin{array}{c} X_{best}\left(t+1\right)+\beta |X_{i,j}\left(t\right)-X_{best}\left(t\right)|\;d>0.1\\ X_{i,j}\left(t\right)+k\left(X_{i,j}\left(t\right)-X_{worst}\left(t\right)\right)\;d<0.1 \end{array}\right.$$

where $X_{best}$ is the current optimal global position and β is the step size of a normally distributed random number with a mean of 0 and variance of 1. $X_{worst}$ is the random initial value when the calculation does not converge, while k represents the step control parameter that determines the direction of parameter calculation, taking a uniform value in the interval [−2, 2]. The symbol d is the expected impedance that matches the imaginary part.

**Figure 8:**
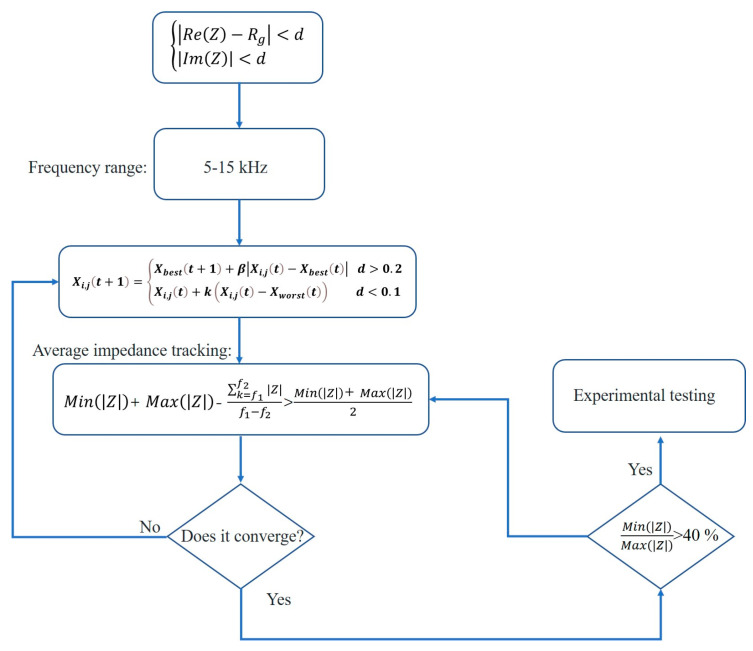
Parameter calculation flowchart.

The impedance matching was designed for the 5–15 kHz frequency band, with a center frequency of 10 kHz. Figure 8 shows a flowchart of the parameter calculation.

After determining the target frequency band, initial value screening was carried out, and the average impedance was used as the objective function for parameter optimization. To achieve the flattest possible matching frequency response, the difference between the maximum and minimum impedances in the frequency band was compared after obtaining the parameters. The final result was output once the defined requirements were met. The impedance-matching network parameters are shown in Table 3.

**Figure 9:**
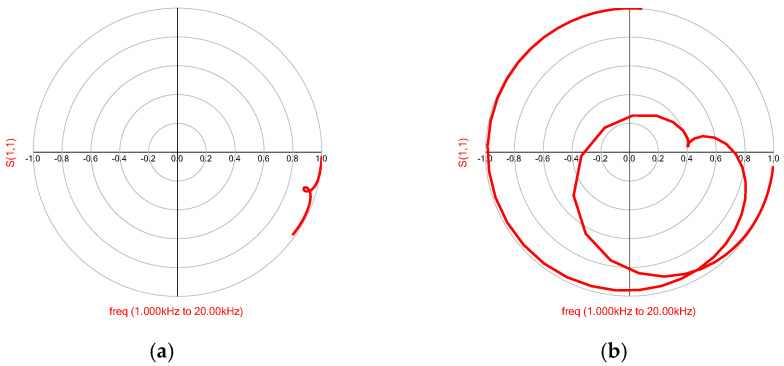
Comparison of reflection coefficient trajectories of transducer equivalent circuit ports. (**a**) Before impedance matching, (**b**) after impedance matching.

Figure 9 shows the polar coordinate diagram of the reflectivity coefficient of the transducer port before and after impedance matching. In Figure 9a, without impedance matching, the transducer shows no reflectivity coefficient trace below 0.4 dB in the target frequency band, indicating significant reactive power loss. This highlights the necessity and effectiveness of the impedance-matching network.

Through optimization of the transducer structure and impedance-matching network parameters, the performance improvement was preliminarily verified.

## 5. Experimental Test Analysis

**Figure 10:**
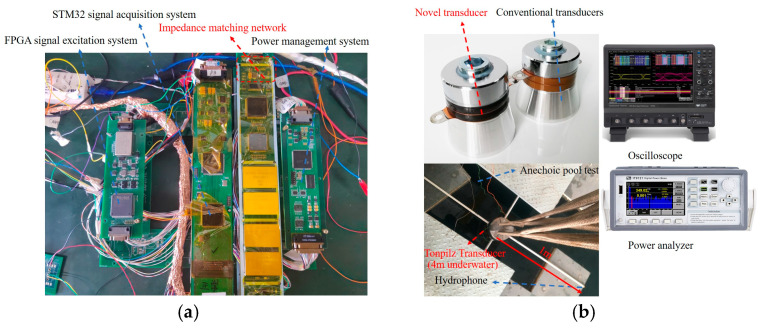
Experimental test environment. (**a**) Transducer excitation and signal acquisition system, (**b**) anechoic pool test.

The experimental setup is shown in Figure 10. As shown in Figure 10a, the field-programmable gate array (FPGA) main control circuit generates high-voltage pulse signals to excite the transducer, while the STM32 main control circuit collects the hydrophone signals. The impedance-matching module is located at the terminal to drive the transducer and radiate acoustic signals. As shown in Figure 10b, experimental tests were conducted on both the novel transducer designed in this study and the conventional Tonpilz transducer in an anechoic pool. The transducer was suspended 4 m underwater, with the hydrophone positioned 1 m away. The hydrophone used in this paper is the TC4034 hydrophone produced by the RESON Company of Slangerup, Denmark. The operating frequency range of the hydrophone is 1 hz~470 kHz, and the receiving sensitivity is: −218 dB ref 1 V/μPa at 250 Hz.

**Figure 11:**
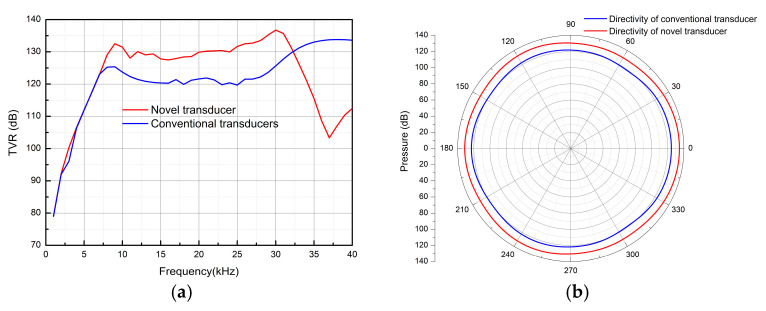
Experimental test environment. (**a**) Transducer TVR experiment test, (**b**) transducer directivity experiment test—15 kHz.

First, the newly designed transducer and a conventional transducer were tested in underwater acoustic experiments. The TVR test results are shown in Figure 11a. It can be observed that the TVR of the new transducer increased by an average of 6 dB in the 10–30 kHz frequency band. Moreover, since the simulation environment did not account for transducer positioning, minor fluctuations appear in the curve. Figure 11b shows the directional test at 15 kHz, indicating that the radiation range of the novel transducer was broader. In summary, the acoustic performance of the newly designed transducer was superior to that of conventional transducers. An oscilloscope and power analyzer were used to collect the excitation voltage, current, phase difference, and hydrophone voltage signals of the transducer.

**Figure 12:**
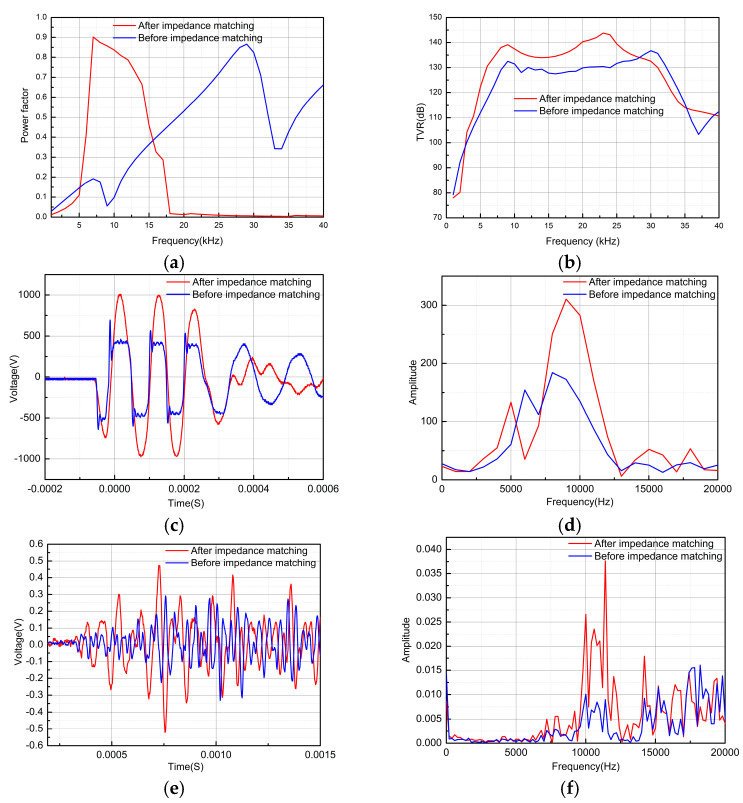
Impedance-matching network excitation experiment. (**a**) Transducer TVR experiment test, (**b**) transducer, (**c**) excitation signal time domain, (**d**) excitation signal frequency domain, (**e**) hydrophone receives the signal time domain, (**f**) hydrophone receives the signal frequency domain.

Subsequently, an impedance-matching excitation test was carried out on the newly designed transducer. Figure 12a shows the power factor of the transducer before and after impedance matching. After impedance matching, the power factor of the transducer end excitation signal is increased by 3.2 times in the target frequency band, and the frequency band width is widened by 1.5 times. Figure 12b shows the TVR test results. The transmitter voltage response increased by an average of 5 dB after impedance matching, indicating improved acoustic field radiation characteristics. Figure 12c shows the excitation voltage signal at the transducer’s terminal. Taking 10 kHz as an example, before impedance matching, significant power reflection at the load end due to impedance mismatch caused numerous peak pulses in the excitation signal, resulting from the static capacitor oscillation discharge of the transducer. After impedance matching, the excitation signal becomes smooth, its amplitude doubles, and a sinusoidal waveform appears, demonstrating that the impedance-matching network provides certain filtering characteristics. Figure 12d shows the excitation signal spectrum, where the energy at the excitation frequency is greatly enhanced. Figure 12e shows the received signal of the hydrophone under 10 kHz excitation, revealing that the received signal increased by 1.7 times after impedance matching. Figure 12f shows the spectrum analysis of the received signal, indicating that the impedance-matching network concentrates the main frequency signal, which is beneficial for subsequent sound field inversion.

Based on the above experimental tests, the sound field radiation characteristics of the novel transducer designed in this paper were 6 dB higher than those of the conventional transducer and were further improved by 5 dB after impedance matching. In addition, the working frequency band can be adjusted according to specific requirements, and the effectiveness of the impedance-matching network was further verified through hydrophone testing.

## 6. Conclusions

To further enhance the acoustic field radiation characteristics of the Tonpilz transducer, we modified its head mass and incorporated a filling structure. The finite element method was used to confirm that steel provided the best material parameters, and the dimensional parameters of the circular table-filling structure were determined through parameter scanning. The average impedance tracking method was used to design the impedance-matching network parameters. Experimental tests verified that the transmitter voltage response of the novel transducer was 6 dB higher than that of a conventional transducer. After impedance matching, the response improved by an additional 5 dB, the power factor increased by 3.2 times, and the frequency band was broadened by a factor of 1.6. The introduction of the filling structure and impedance-matching network provides greater flexibility in Tonpilz transducer design, allowing for quick adjustments of the operating frequency band or focusing on a specific frequency for maximum power output, thereby expanding the possibilities for underwater acoustic detection.

## Figures and Tables

**Table 1:** Transducer material parameters.

**Section of the Transducer**	Material	$\rho (kg/m^{3})$	c(m/s)	$d_{33}(C/m)$	$S_{33}(N^{2}/m)$
Tail mass and bolt	Steel	7850	5050	-	-
Piezo ceramic stack	PZT-5A	7500	2930	$28\times 10^{-11}$	$S_{33}^{E}=15\times 10^{-12}$ $S_{33}^{D}=80\times 10^{-13}$
Head mass	Aluminum	2700	5100	-	-
New structure	Epoxy resin or steel	-	-	-	-

**Table 2:** Basic parameters of filling material.

Material	$\rho (kg/m^{3})$	$Poisson^{’}s\;$ Ratio	E(Pa)
Steel	7850	0.28	205 × 10^9^
Aluminum	2700	0.33	70 × 10^9^
Epoxy resin	1500	0.32	10 × 10^9^

**Table 3:** Impedance-matching network components.

Impedance-Matching Component	Value
$L_{s}$	55.2 mH
$L_{p}$	2.16 mH
$C_{p1}$	0.88 nF
$C_{p2}$	5.32 pF

## Data Availability

The original contributions presented in this study are included in the article. Further inquiries can be directed to the corresponding authors.
